# *Salmo salar* fish waste oil: Fatty acids composition and antibacterial activity

**DOI:** 10.7717/peerj.9299

**Published:** 2020-06-19

**Authors:** Luigi Inguglia, Marco Chiaramonte, Vita Di Stefano, Domenico Schillaci, Gaetano Cammilleri, Licia Pantano, Manuela Mauro, Mirella Vazzana, Vincenzo Ferrantelli, Rosalia Nicolosi, Vincenzo Arizza

**Affiliations:** 1 STEBICEF, University of Palermo, Palermo, Italy, Italy; 2 Istituto Zooprofilattico della Sicilia “A.Mirri”, Palermo, Italy, Italy

**Keywords:** Waste oil, Polyunsaturated fatty acid, Minimum inhibitory concentration, Salmo salar, Fish oil

## Abstract

**Background and aims:**

Fish by-products are generally used to produce fishmeal or fertilizers, with fish oil as a by-product. Despite their importance, fish wastes are still poorly explored and characterized and more studies are needed to reveal their potentiality. The goal of the present study was to qualitatively characterize and investigate the antimicrobial effects of the fish oil extracted from *Salmo salar* waste samples and to evaluate the potential use of these compounds for treating pathogen infections.

**Methods:**

*Salmo salar* waste samples were divided in two groups: heads and soft tissues. Fatty acids composition, and in particular the content in saturated (SAFAs), mono-unsaturated (MUFAs) and Polyunsaturated (PUFAs) fatty acids, was characterized through GC/MS Thermo Focus GC-DSQ II equipped with a ZB-5 fused silica capillary tubes column. The antimicrobial activity of the salmon waste oils was evaluated through the Minimum Inhibitory Concentration assay and the antibiotics contamination was determined by Liquid Chromatography with tandem Mass Spectrometry (LC-MS/MS) analysis. All experiments were done at least in triplicate.

**Results:**

GC/MS analysis has shown the specific fatty acid composition of the salmon waste oils and their enrichment in MUFAs and PUFAs, with special reference to omega-3, -6, -7, -9 fatty acids. Furthermore, our study has highlighted the antimicrobial activity of the fish waste oil samples against two Gram+ and Gram- bacterial strains.

**Conclusions:**

These data confirm that the fish waste is still quantitatively and qualitatively an important source of available biological properties that could be extracted and utilized representing an important strategy to counteract infective diseases in the context of the circular economy.

## Introduction

Fish by-products are generally used to produce fishmeal or fertilizers, with fish oil as a by-product. Anyway, fish wastes are an enormous potential source of useful molecules, such as bioactive peptides, enzymes, antimicrobial components and polyunsaturated fatty acids (PUFA) (Ghaly et al., 2013). Despite their importance, fish wastes are still poorly explored and characterized and more studies are needed to reveal their potential.

Polyunsaturated fatty acids (PUFAs) are key constituents of the cell membranes. For this reason they are important regulators of the membrane fluidity, cell signalling, gene expression and cellular functions, and also represent important substrates for lipid mediators synthesis (Parolini, 2019). Low serum levels of ω-3 polyunsaturated fatty acids (PUFAs) have been associated to an increased incidence of cardiovascular disease and higher mortality rate. Furthermore, pre-clinical and clinical studies have shown that the addition of ω-3 PUFAs to daily diet can prevent and attenuate lipid accumulation, vascular inflammation and macrophage recruitment, which are among the major causes of the atherosclerosis plaque formation process (García-Hernández et al., 2013; Corsi, Momo Dongmo & Avallone, 2015; Yagi et al., 2017; Leshno et al., 2018). The American Institute of Medicine, Food and Nutrition Board recommended daily Adequate Intakes (AIs) of Omega-3s (Medicine, 2005) which, unfortunately, are often not met with the daily diet.

The role of PUFAs as pro- or anti-inflammatory molecules has been largely discussed (Fischer & Weber, 1983; Heidel et al., 1989; Hawkes, James & Cleland, 1992; Moreno, 2009; Simonetto et al., 2019). Lipid mediators derived from the ω-6 PUFA are involved in inflammation at different stages. For example, the ω-6 PUFA arachidonic acid is the precursor of prostaglandins, thromboxanes and pro-inflammatory leukotrienes. Instead, ω-3 PUFAs exhibit anti-inflammatory properties by competing with ω-6 PUFAs, altering the membrane phospholipid amount of arachindonic acid and reducing the production of pro-inflammatory eicosanoids. Furthermore, ω -3 PUFAs promote the resolution of inflammation through the synthesis of lipid mediators, including resolvins, protectins and maresins, which are known to be “specialized pro-resolving mediators” (SPMs) (Serhan, 2014).

PUFAs and free fatty acids (FFA) have also been studied for their antimicrobial activity which is characterized by a broad spectrum of activity and the lack of classical resistance mechanisms (Desbois & Smith, 2010; Desbois, 2012; Desbois & Lawlor, 2013). In a recent paper (Chanda et al., 2018), different hypothesis were proposed as PUFAs antimicrobial mode of action which include disruption of intercellular communication, interruption of ATP production, alteration of membrane properties, disruption of fatty acids synthesis, affecting the electron transport system and increasing the number of membrane pores (Zheng et al., 2005; Carballeira, 2008). In addition, as explained elsewhere (Desbois & Smith, 2010), FFAs can also impair *Staphylococcus aureus* skin colonization through stratum corneum acidification (Fluhr et al. 2001; Takigawa et al., 2005). FFAs may also impair the expression of virulence factors, which are necessary for the establishment of infection, and inhibit the cell-to-cell signalling, thus preventing initial bacterial adhesion and subsequent biofilm formation.

Marine organisms are a very important source of antimicrobial agents (Richards et al., 2001; Schillaci et al., 2010; Schillaci et al., 2013; Spinello et al., 2018; Vazzana et al., 2018; Núñez Acuña et al., 2018) and, among them, the PUFAs are normally present at high levels in *Salmo salar* (Linder, Fanni & Parmentier, 2005; Morais et al., 2009). Different studies have shown the antimicrobial activity of the salmon PUFAs against Gram-positive bacteria due to their specific components such as eicosapentaenoic acid (Desbois, Mearns-Spragg & Smith, 2009; Desbois & Lawlor, 2013), docosahexaenoic acid (Coonrod, 1987; Feldlaufer et al., 1993; Gladyshev et al., 2009), γ-linolenic acid (Asthana et al., 2006) and dihomo- γ-linolenic acid (Feldlaufer et al., 1993).

It is well known that among fishes farmed in Europe, the Atlantic salmon (*Salmo salar*) is one of the most important aquaculture species. Norway, Scotland and Ireland are the three major producers of salmon, which, in Europe, represents an important resource in terms of production and economic value. Fish waste from salmon can reach up to 40% of the total weight of the animal and is mainly composed of bones, head and offal.

Even if the most important source of fatty acids is the adipose tissue interspersed in the muscular fibers, it is still possible to obtain valuable quantities of PUFAs from the fish waste due to their large amount. Furthermore, the possibility to use the fish leftovers and to produce, from them, high value products, represents an important step in the circular economy.

Thus, the aim of the present research was to characterize, by qualitative point of view, and to investigate, for the first time, the antimicrobial effects of the fish oil extracted from the market salmon waste through an unbiased approach regard fishing or farming source and condition. Furthermore the potential use of these compounds, for treating Gram + and Gram- bacterial infections, was evaluated through the determination of minimum inhibitory concentrations against *S. aureus* and *Pseudomonas aeruginosa*, two relevant pathogens cause of polymicrobial infections (Serra et al., 2015) and included in the global priority list of antibiotic-resistant bacteria from WHO/OMS (Tacconelli et al., 2017). In addition, we tried to understand if the antimicrobial activity could be due to any chemical contaminants present in the samples.

## Materials and Methods

### Salmon sample collection, storage and fish oils extraction

Ten animals were collected from small fish markets of Palermo (Italy) and utilized for the experiments. The animal size is the commercial size (about 2 years old and 3–5 Kg). Raised farm and feed composition are not available. Salmon wastes were transported at +4 °C to the laboratory for the analysis. Fish wastes were divided into two groups which comprise heads and soft tissues that were homogenized and stored at −20 °C until the time of analysis. Homogenate samples were diluted in distilled water (1:1) and heated at 90 °C for 1 h, to coagulate proteins and increase oil release from samples. This method, well established and utilized, was chosen for simplicity and laboratory cost effectiveness. After heat treatment, the homogenate was filtered to obtain the liquid fraction that was centrifuged at 10,000 g a 4 °C to separate the aqueous fraction from the liposoluble component corresponding to the fish oil.

### GC/MS analysis and identification of the components of *Salmo salar* PUFAs

Fatty acids were analyzed using GC-MS method after extraction and hydrolysis of triacylglycerols. 0.1 g of oil samples were diluted in 1 ml of n-heptane and manually agitated for 10 s, followed by the addition of 0.1 ml of 2N KOH in MeOH solution and mixed in a vortex. When the solution turned clear, 500 µl of upper phase, containing fatty acid methyl esters was diluted with *n*-heptane to a final volume of 1 ml. For the separation and analysis of the fatty acid methyl esters, Thermo Scientific ISQ™ 9000 Quadrupole GC-MS System in EI (Electron Ionization) mode, working in full scan was used. The capillary column used was a ZB-WAX (30 m ×  0.25 mm i.d., film thickness 0.25 µm, (Phenomenex, Italy). The oven temperature was programmed so that column temperature started at 80 °C, increased at 15 °C/min to 250  °C and held for 8 min under isothermal conditions. Helium was used as the carrier gas at a flow rate of one mL/min. A sample of 1 µl was injected with a split ratio of 1:100. Mass spectroscopy conditions: The ion source temperature was 260 °C, the MS transfer line temperature was 265  °C and injector temperature was 250 °C. Ionization voltage was 70 eV and the mass range scanned was 35–550 m/z. Using Thermo Scientific Xcalibur Data system software for Windows peak areas were determined and identified by comparison of retention times with those of a FAMEs standard mix (Supelco 37 Component FAME Mix, CRM47885 Sigma-Aldrich) separated under the same chromatographic conditions. Triplicate analyses were prepared for each dried sample, and analysed FAMEs were expressed in percentage.

### Samples extraction and LC-MS/MS analysis

Samples were subjected to extraction and antibiotics determination (Quinolonics, Fluoroquinolonics, Penicillins, Tetracyclines, Macrolides, Sulfamidic and Sulfonamides) following the protocol reported in a previous paper (Cammilleri et al., 2019b). The analyses were performed on a Thermo Fischer UHPLC system (Thermo Fisher Scientific, California, USA) consisting of an ACCELA 1250 quaternary pump and an ACCELA autosampler. A Thermo Scientific Hypersil Gold reversed-phase UHPLC column (50 mm, 2.1 mm ID, 1.9 µm) was used for the chromatographic separation. The LC eluents were water (A) and acetonitrile (B), containing 0.1% (v/v) formic acid. The gradient started with 95% eluent A for 1.0 min, a linear variation to 10% A in 6.0 min; these conditions were maintained for 3.0 min. The system returned to 95% A in 0.5 min and was re-equilibrated for 5 min. The column temperature was 30 °C and the sample temperature was kept at 6 °C. The flow rate was 0.4 ml/min and the injection volume was 5 µl. A triple quadrupole TSQ Vantage (Thermo Fisher Scientific, California, USA) in positive electrospray ionization mode (ESI) mass spectrometer was used. The product ion scans of each analyte were performed by direct infusion (10 µl/min) of 1 mg l^−1^ individual standard solutions with the built-in syringe pump through a T-junction, mixing with the blank column eluate (200 µL/min). The ESI parameters optimized were as follows: capillary voltage 4.5 kV; capillary temperature 310 °C; vaporizer temperature 150 °C; sheath and auxiliary gas pressure were fixed at 40 and 15 (arbitrary unit), respectively. The collision gas was argon at 1.5 mTorr and peak resolution of 0.7 FWHM was used on Q1 and Q3. The scan time for each monitored transition was 0.02 s and the scan width was 0.02 m/z. Acquisition data were recorded and elaborated using Xcalibur ^TM^ version 2.1.0.1139 software from Thermo. The method was validated according to the parameters described by EU Commission Decision 2002/657/EC. All experiments were done at least in triplicate.

### Minimum inhibitory concentration determination

Minimum inhibitory concentrations (MICs) of the fish oils from *Salmo salar*, head and muscle, were evaluated using an already described micromethod (Schillaci et al., 2010; Schillaci et al., 2013; Schillaci et al., 2014). A series of solutions were prepared with a range of concentrations from 50 to 0.75 % v/v (obtained by two-fold serial dilution). The serial dilutions were made in Mueller-Hinton Broth (MHB) in a 96-wells plate, starting from a stock solution of 1 mg/mL in NaCl 0.9% w/v. To each well, 10 µL of a bacterial suspension from a 24 h culture containing ∼10^6^ cfu/mL was added.

The plate was incubated at 37 °C for 24 h; after this time, the MICs were determined by a microplate reader (Glomax Multidetection System TM297 Promega, Milano Italy) as the lowest concentration of compound whose Optical Density (OD), read at 570 nm, was comparable with the negative control wells (broth only, without inoculum). Positive controls are instead the growth control, bacterial inoculum in the medium without any inhibitor. The antimicrobial properties were determined on Gram-positive bacterial reference strains *Staphylococcus aureus* (ATCC 25923; ATCC 6538) and on Gram-negative strains *Pseudomonas aeruginosa* (ATCC 15442; ATCC 9027). Each assay was performed in triplicates and repeated at least twice.

## Results

### *Salmo salar* waste oils characterization by GC/MS analysis

The fish oil fatty acid compositions extracted from head and soft tissue were characterized, qualitatively, by GC/MS. The analysis, whose results are listed in Tables 1A and 1B, showed the salmon fatty acid composition and the percentage of the different unsaturated (UFAs) and saturated fatty (SFAs) acids both in the head and in the soft tissue respect to the total. In particular, the fish waste oil from the heads was composed of the 84% of UFAs and the 16% of SFAs while the fish waste oil from the soft tissue was composed of 83% of UFAs and the 17% of SFAs.

Among the unsaturated fatty acids, the GC/MS has highlighted the presence of the monounsaturated acids ω-9 oleic (C18:1 Δ^9^), which percentages were of 53.58% in the head oil and 39.47% in the soft tissue oil, ω-9 gondoic (C20:1 Δ^11^), which percentages were of 5.75%, in the head oil and 4.02% in the soft tissue oil and ω-7 Palmitoleic (C16:1 Δ^9^), 3.24% in the head oil and 1.39% in the soft tissue oil. The polyunsaturated acids were mainly composed of ω-3 α-Linolenic (C18:3 Δ^9,12,15^ ), 5.91% in the head oil and 4.46% in the soft tissue oil and ω-6 Linoleic (C18:2 Δ^9,12^), 15.43% in the head oil and 14.56% in the soft tissue oil.

**Table 1:** Salmo salar fatty acid characterization. Fatty acid composition of the oil extracted from wastes of Salmo salar head samples (A) and soft tissue samples (B). Values are reported as relative percentages and are means ±standard deviations. All experiments were done at least in triplicate.

**(A)**	Salmon head oil			
**IUPAC**	**NAME**	***ω*-Group**	**R.T.**	**RELATIVE % ± D.S.**
C14:0	Myristic acid	–	10.10	2.28 ± 0.04
C16:0	Palmitic acid	–	10.84	11.44 ± 0.16
C16:1 Δ^9^	Palmitoleic acid	*ω*-7	10.91	3.24 ± 0.24
C18:0	Stearic acid	–	11.52	2.34 ± 0.12
C18:1 Δ^9^	Oleic acid	*ω*-9	11.56	53.58 ± 0.72
C18:2 Δ^9,12^	Linoleic acid	*ω*-6	11.70	15.43 ± 0.37
C18:3 Δ^9,12,15^	α-Linolenic acid	*ω*-3	11.89	5.91 ± 0.10
C20:1 Δ^11^	Gondoic acid	*ω*-9	12.34	5.75 ± 0.99

**(B)**	Salmon soft tissue oil			
**IUPAC**	**NAME**	***ω*-Group**	**R.T.**	**RELATIVE % ± D.S.**
C14:0	Myristic acid	–	9.59	2.56 ± 0.29
C14:1 Δ^9^	Myristoleic acid	–	9.74	0.03 ±0.01
C15:0	Pentadecanoic acid	–	10.05	0.14 ±0.12
C16:0	Palmitic acid	–	10.33	9.57 ±0.38
C16:1 Δ^9^	Palmitoleic acid	*ω*-7	10.49	1.39 ±1.59
C16:2 Δ^9,12^	9,12-Hexadecadienoic acid	–	10,65	0.13 ±0.06
C17:0	Margaric acid	–	10.73	0.2 ±0.03
C17:1 Δ^10^	cis-10- Heptadecenoic acid	*ω*-7	10.80	0.16 ±0.14
C17:1 Δ^8^	Heptadecenoic	*ω*-9	10.85	0.13 ±0.04
C18:0	Stearic acid	–	10.95	0.54 ±0.6
C18:1 Δ^9^	Oleic acid	*ω*-9	11.05	39.47 ±2.57
C18:2 Δ^9,12^	Linoleic acid	*ω*-6	11.25	14.56 ±0.19
C18:2 Δ^12,15^	12,15-Octadecanoic acid	*ω*-3	11,38	0.09 ±0.01
C18:3 Δ^6,9,12^	*γ*-Linolenic acid	*ω*-6	11,45	2.13 ±3.49
C18:3 Δ^9,12,15^	*α*-Linolenic acid	*ω*-3	11,50	4.46 ±3.2
C18:4 Δ^6,9,12,15^	Stearidonic acid	*ω*-3	11,62	0.59 ±0.5
C20:0	Eicosanoic acid	–	11,81	2.25 ±3.36
C20:1 Δ^11^	Gondoic acid	*ω*-9	11,87	4.02 ±3.45
C20:2 Δ^8,11^	8,11-Eicosenoic acid	–	12,01	0.58 ±0.91
C20:2 Δ^11,14^	cis-11,14-Eicosadienoic acid	*ω*-6	12,06	1.27 ±0.84
C20:3 Δ^8,11,14^	8,11,14-Eicosatrienoic acid	*ω*-6	12,16	0.39 ±0.26
C20:3 Δ^11,14,17^	11,14,17-Eicosatrienoic acid	*ω*-3	12,28	0.82 ±0.08
C20:4 Δ^8,11,14,17^	all-cis 8,11,14,17-Eicosatetraenoic acid	–	12,36	1.53 ±0.80
C20:5 Δ^5,8,11,14,17^	Eicosapentaenoic acid (EPA)	*ω*-3	12,45	1.90 ±1.42
C22:0	Docosanoic acid	–	12,63	1.57 ±2.64
C22:1 Δ^13^	Erucic acid	*ω*-9	12,69	3.33 ±2.88
C22:2 Δ^13,16^	cis-13,1 6-Docosanoic acid	*ω*-6	12,99	0.26 ±0.12
C22:5 Δ^4,7,10,14,16^	4,7,10,13,16-Docosapentaenoic acid	*ω*-6	13,34	0.45 ±0.61
C22:5 Δ^7,10,13,16,19^	Clupanodonic acid (OPA)	–	13,51	2.03 ±1.17
C22:6 Δ^4,7,10,13,16,19^	Docosahexaenoic acid (DHA)	*ω*-3	13,65	2.78 ±2.04
C24:0	Tetracosanoic acid		13,75	0.12 ±0.10
C24:1 Δ^15^	Nervonic acid	*ω*-9	13,88	0.29 ±0.24

The saturated fatty acids (SFA) represented the remaining part of the lipid fraction constituted by myristic acid (C14:0), 2.28% in the head oil and 2.56% in the soft tissue oil, palmitic acid (C16:0), 11.44% in the head oil and 9.57% in the soft tissue oil and stearic acid (C18:0), 2.34% in the head oil and 0.54% in the soft tissue oil. Furthermore, eicosanoic acid (C20:0) and docosanoic acid (C22:0) were detected only in the soft tissue oils with the relative ratios of 2.25% and 1.57% respectively.

### Antimicrobial activity of the *Salmo salar* fish waste oils

The antimicrobial activity of the oils extracted from the fish waste, both the head and the soft tissue, was evaluated through the determination of the MIC values against two reference strains of Gram-positive (*S. aureus*) and Gram-negative (*P. aeruginosa*) bacteria. Table 2 summarizes the results of the experiment in which the antimicrobial efficacy the fish waste oils has been determined. In fact, the fish oils extracted from the head and from the soft tissue were shown to inhibit the growth of the tested microorganisms at a concentration of 25% (v/v) and 12.5% (v/v) respectively.

**Table 2:** Salmon waste oils antibacterial activity. Antimicrobial activity (MIC) of the fish oil extracted from salmon head and soft tissue samples against two Gram+ and Gram- bacterial strains. Values are expressed as volume percentages (%v/v).

		MIC (%v/v)	
Bacterial strains		Soft tissue	Head
Gram-	*P. aeruginosa* ATCC 9027	12.5	25
	*P. aeruginosa* ATCC15442	12.5	25
Gram+	*S. aureus* ATCC 6538	12.5	25
	*S. aureus* ATCC 25923	12.5	25

### Antibiotics contaminants evaluation

To assess the possibility of antibiotics contaminants in the fish waste oil samples, an LC-MS/MS was performed. The validation of the method produced satisfactory results in terms of linearity (r^2^ > 0.996 for all the analytes examined), accuracy and precision of intra-day and inter-day analysis, with relative standard deviation (RSD) values within 10%. The trueness values obtained were in the range of 86–92%. The residues of antibiotics found in the samples examined are shown in Table 3.

**Table 3:** Antibiotics contaminants determination in salmon waste oil. LC-MS/MS antibiotics determination in the fish oil extracted from salmon head and soft tissue samples. Values are expressed as g/Kg. N/D, not detected.

**Functional groups**	**Antibiotic**	**Head oil (µg/Kg)**	**Soft tissue oil (µg/Kg)**
Sulfonamide	Sulfaguanidine	6.48	6.54
	Sulfamerazine	N/D	0.72
Fluoroquinolon	Ofloxacin	2.32	N/D
	Ciprofloxacin	3.85	N/D
	Lomefloxacin	1.83	N/D
	Enrofloxacin	N/D	0.17
Sulfamidic	Sulfachinossalin	2.19	N/D
Quinolon	Nalidixic Acid	2.04	1.97
Macrolides	Oleandomycin	4.58	4.83
	Tylosina	4.48	N/D
β-lactam	Penicillin G	9.29	31.29
	Penicillin V	N/D	50.73
	Oxacillin	N/D	34.34
	Nafcillin	21.31	151.04

Among 53 antibiotics tested, both types of sample examined showed the simultaneous presence of 4 antibiotics classes, Quinolonics, β-lactams, Macrolides and Sulfonamides while Fluoroquinolons and Sulfamidics were highlighted only in the head oil sample. No tetracycline residues were found. β-lactams were found to be present at the highest concentrations.

## Discussion

Despite its potential value, fish waste actually represents a significant cost for fish industries and markets. This huge mass is normally discarded but could represent an important source of bioactive compounds for pharmaceutical, cosmetic, nutrition and biotechnological applications. Molecules such as proteins (i.e., enzymes and collagen), lipids, protein hydrolysates, astaxanthin, chitin (Caruso, 2015) can be extracted and utilized. Fish oil, enriched in Polyunsaturated Fatty Acids (PUFAs), is still another important source of high quality bioactive molecules that could be extracted from the fish waste.

*Salmo salar* fish waste can represent up to 30–50% of the total weight of the animal (Torrissen et al., 2011; He, Franco & Zhang, 2011; Opheim et al., 2015; Dinh et al., 2018). It is well known that the salmon oil has important features in terms of PUFAs and is a rich source of omega-3 fats. In our study we have confirmed the presence of PUFAs in the fish waste both in the oil extracted from head and soft tissues and we have shown how these PUFAs are characterized by the presence of omega-3 and omega-6 fatty acids (Tables 1A and 1B). The most abundant omega-3 found in the fish waste oil was the linolenic acid, which represent 5.91% in head oil and 4.46% in soft tissue oil. The α-Linolenic acid is really important in the human diet due to its role as substrate for the synthesis of eicosapentaenoic acid (EPA) and docosahexaenoic acid (DHA), which confer unique biophysical properties to cell membranes and are necessary for tissue functions (Burdge & Calder, 2006). Linoleic acid was found to be the most abundant omega-6 fatty acid both in head oil (15.43% ± 0.47%) and in soft tissue oil (14.56% ± 0.19%). This fatty acid is known for its important physiological role, as a constituent of acylglycosyl ceramides, in maintaining the integrity of the water permeability barrier of the skin (Sanders, 2016). The omega-6 linoleic acid is also the precursor of the arachidonic acid, which, in turn, is the major precursor of the eicosanoids, such as prostaglandins, thromboxanes, prostacyclins, leukotrienes and anandamides, which control a large number of physiological processes. Furthermore, arachidonic acid regulates the membrane fluidity, modulates the function of specific membrane proteins involved in cellular signalling and act maintaining cell and organelle integrity and vascular permeability (Tallima & El Ridi, 2018). Arachidonic acid also affects neuronal excitability and synaptic transmission through acting on voltage-gated ion channels and the accumulation of unesterified arachidonic acid compromises cell survival via induction of apoptosis. Omega-9 oleic acid was the most abundant fatty acid found both in the head oil (53.58% ± 0.72) and in soft tissue oil (39.47% ± 2.57%). This molecule, that is a monounsaturated fatty acid, has been shown to exert many biological functions such as regulation of plasma lipid and lipoprotein concentrations, modification of coagulation properties, improvement of glucose homeostasis, and attenuation of inflammation and oxidative stress (Lopez et al., 2010). Furthermore oleic acid has been shown to inhibit tumour cell proliferation in a dose- and time-dependent manner, inducing cell cycle G0/G1 arrest, increasing the apoptosis and the expression of p53 and cleaved caspase-3, and decreasing the expression of CyclinD1 and Bcl-2 (Jiang et al., 2017).

Fatty acids also exert an important role against microorganism infections. Pathogenic agents can cause infections in different ways, such as through the production of virulence factors and biofilms formation, (Beceiro, Tomás & Bou, 2013; Schroeder, Brooks & Brooks, 2017). *In vitro* and *in vivo* studies have demonstrated that omega-3 fatty acids, and in particular linolenic acid and its derivatives, used alone or in combination with conventional antibiotics, possess antimicrobial properties (Chanda et al., 2018). Omega-6, -7, -9 fatty acids, such as γ-linolenic, linoleic, arachidonic, palmitoleic and oleic acids, their ethyl esters and methyl esters, were also shown to be effective against various microorganisms (Huang, George & Ebersole, 2010). The mechanism of action that would explain the antimicrobial properties of fatty acids could be the alteration of cell membrane hydrophobicity, charge and integrity, which result in electron leakage and subsequent cell death (Desbois & Smith, 2010; Lopez-Romero et al., 2015; Calo et al., 2015). Furthermore fatty acids can contribute to bacterial death through cell lysis, inhibition of enzyme activity, impairment of nutrient uptake and the generation of lethal oxidation products (Desbois & Smith, 2010).

In our research, the antimicrobial properties of the salmon head and soft tissue waste oils were tested against two important Gram positive and Gram negative pathogens and the results showed a MIC of 25% and 12.5% (v/v) respectively (Table 2). These data seem to indicate that the fish oil extracted from the waste is still active against tested microorganisms and can act independently from the bacterial wall type. Furthermore, the antimicrobial activity of the waste oil extracted from the salmon soft tissue seems to be more efficient than head oil against the two bacterial strains utilized. This difference could be probably due to the different fatty acid composition of the two tissue sources (Tables 1A and 1B). In fact, comparing the fatty acids composition in the two samples, SUFAs, MUFAs and PUFAs were highest in the oil extracted from soft tissue. These data seem to be in accordance with similar results obtained in other fishes (Li, Sinclair & Li, 2011; Hong et al., 2015).

Our research was performed through an unbiased approach regard fishing or farming source and condition. However, it is legitimate to ask whether farming conditions, and in particular fish diet, could alter the fish oil composition. Recently studies have shown that alternative salmon feeds can increase the salmon oil concentration but not, at least completely, its composition (Ruyter et al., 2019; Bruni et al., 2020)

Almost the totality of the Salmon sold in Italian fish markets is produced through aquaculture methods. The importance of food safety is crucial for fish farm and the potential hazards include dangerous chemicals and microorganisms. The former could be accumulated by the fish, especially in their fat tissues, from the aquaculture environment, the feed and residues from veterinary prophylaxis, the latter are represented by parasites, viruses and bacteria that may be harmful for humans (Fairgrieve & Rust, 2003; Estévez et al., 2018; Ben Hamed et al., 2018; Gjessing et al., 2019; Feist et al., 2019). In addition seafood produced through aquaculture can be contaminated by different types of toxic substances from natural and/or anthropogenic origins due to both indirect and direct pollution from continental human activities (Fremy & Bordet, 2002; Chiesa et al., 2019; Quiñones et al., 2019; Heldal et al., 2019). Aquaculture fish management practices can represent an important stressing factor for fishes, in particular for salmon, and could result in high mortalities leading to significant economic loss for producers (Wilson et al., 2009; Sudheesh et al., 2012; Overton et al., 2019). In fact, stressors like handling, stripping of brood stock, antimicrobial treatments, vaccination, temperature, crowding, starvation and transport can result in an increase of a number of diseases evaluable through the measure of the levels of cytokines, heat shock proteins (HSP), corticosteroid hormones, immunoglobulin and immune cells levels, haematological parameters (Gabriel & Akinrotimi, 2011; Cordero et al., 2016; Rehman et al., 2017; Parisi et al., 2017; Chiaramonte et al., 2019; Cammilleri et al., 2019a; Inguglia et al., 2020; Vazzana et al., 2020). Moreover, salmon, which are usually farmed in crowded conditions, are easily targeted by infective pathologies (Poppe, Barnes & Midtlyng, 2002; Håstein, 2004; Bang-Jensen, Gu & Sindre, 2019). Over the past years, fish farms, and in particular that of salmon, have increased their productivity in parallel with the growth of the use of substances used to prevent and treat microbial and bacterial disease, such as antibiotics (Miranda, Godoy & Lee, 2018). We must say that other solutions are being tried, such as vaccines and immunostimulants (Eslamloo et al., 2017; Meza et al., 2019; Xue et al., 2019; Chalmers et al., 2020), in order to limit the use of antibiotics (Gravningen, Sorum & Horsberg, 2019) but they still remain a largely utilized solution expecially in Non-European countries. Specific data about antibiotics in the aquaculture industry are not easy to report due to the different current laws of the involved countries. For this reason data are often unavailable or unattainable (Heuer et al., 2009; Romero, Gloria & Navarrete, 2012; Miranda, Godoy & Lee, 2018). However, based on available data, the qualities and quantities of the used antibiotics are variable. For example, among the world countries with the highest rates of aquaculture antibiotic use (Van Boeckel et al., 2015) there are Chile and Vietnam. In Norway, the antibiotics amount used in aquaculture, decreased enormously in the last years but still present, is of 1g/ton of farmed Salmon while in Vietnam the utilized amount is of 700 g/ton of farmed shrimp (Smith, 2008; Bang-Jensen, Gu & Sindre, 2019).

Considering this information, we performed the LC-MS/MS analysis to exclude the involvement of aquaculture contaminants in the biological antibacterial activity of the fish waste oil (Table 3). The analysis has shown the presence of traces of molecules that, anyway, are considerably under the maximum residue limits indicated by European law (REGULATION, 2009; Commission Regulation (EU), 2009). Furthermore, the small drug amounts highlighted by the experiment would not seem explain the MIC values observed. However, we cannot completely exclude the hypothesis of a contribute of this molecules to the antibacterial properties of the fish waste oil and further analysis are needed to totally exclude this possibility.

## Conclusions

The present research have shown, through GC-MS analysis, the specific composition of the fish waste oil extracted from different discarded parts of the *Salmo salar* present in Italian fish markets. The analysis has also highlighted the oil enrichment in polyunsaturated fatty acids and, among them, in omega-6, -7 and -9 fatty acids. In addition, the MIC experiments have revealed the antibacterial activity of the extracted Salmon waste oil

These data confirm that the fish waste is still quantitatively and qualitatively an important source of available biological properties that could be extracted and utilized representing an important strategy to counteract infective diseases in the context of the circular economy.

##  Supplemental Information

10.7717/peerj.9299/supp-1 Supplemental Information 1 GC/MS raw data exported from the Xcalibur software utilized for fish soft tissue oil data analyses and preparation of Table 1

10.7717/peerj.9299/supp-2 Supplemental Information 2 GC/MS raw data exported from the Xcalibur software utilized for fish head oil data analyses and preparation of Table 1

10.7717/peerj.9299/supp-3 Supplemental Information 3 GC/MS technical replicate raw data of fish soft tissue oil, exported

10.7717/peerj.9299/supp-4 Supplemental Information 4 Raw data for antibiotic (LC-MS/MS) analysis

10.7717/peerj.9299/supp-5 Supplemental Information 5 Raw data for minimal inhibitory concentration of the waste fish oils

## Additional Information and Declarations

### Data Availability

The following information was supplied regarding data availability: Raw data are available in the Supplementary Files.
